# Efficacy of probiotic supplementation in preventing *Clostridioides difficile* infection: an umbrella review of systematic reviews and meta-analysis

**DOI:** 10.3389/fnut.2026.1699223

**Published:** 2026-03-09

**Authors:** Wenci Chen, Xianjuan Pan, Jing Ji, Zhenhua Wu, Xinxin Lin

**Affiliations:** 1 Encephalopathy and Rehabilitation Center, The Second Affiliated Hospital of Zhejiang Chinese Medical University, Hangzhou, Zhejiang, China; 2 Department of Rehabilitation, Wenzhou Hospital of Integrated Traditional Chinese and Western Medicine, Wenzhou, Zhejiang, China; 3 Department of General Practice, The Second Affiliated Hospital, Yuying Children's Hospital of Wenzhou Medical University, Wenzhou, China

**Keywords:** *Clostridioides difficile* infection, meta-analysis, probiotics, systematic review, umbrella

## Abstract

**Background:**

*Clostridioides difficile infection* (CDI) is a significant healthcare-associated infection. Probiotics have been proposed as a preventive strategy. This umbrella review synthesizes evidence from meta-analyses on the efficacy of probiotics in preventing CDI.

**Methods:**

A comprehensive systematic search was conducted in PubMed, Scopus, Web of Science, Embase, and the Cochrane Library up to December 2025. To manage overlap, a single primary systematic review was selected per outcome. The methodological quality of included reviews was assessed using AMSTAR-2, and the certainty of evidence was graded. Pooled effect sizes were calculated using a random-effects model.

**Results:**

Sixteen systematic reviews and meta-analyses were included. The pooled relative risk (RR) from primary reviews indicated that probiotics significantly reduced CDI risk (RR = 0.37; 95% CI: 0.32 to 0.42; *I*^2^ = 0%). Multi-strain probiotics and formulations containing *Saccharomyces boulardii* showed significant benefits. The quality of evidence ranged from moderate to low, and overlap among primary studies was minimal (Corrected Covered Area = 15%).

**Conclusion:**

Probiotic supplementation is associated with a reduced risk of CDI. However, given the variable methodological quality of the underlying evidence, these findings should be interpreted with caution. Population-specific and strain-specific effects require further investigation.

## 1 Introduction

*Clostridioides difficile infection (CDI)* is a common healthcare-associated challenge, primarily linked to antibiotic use (1, 2). It frequently manifests as diarrhea in hospitalized patients (3). Antibiotic treatment disrupts the gut microbiota, facilitating the development of CDI (4). Evidence indicates that CDI is associated with a wide range of health consequences, including severe diarrhea and systemic infection (5). It is a multifaceted disorder with broad clinical and socio-economic impacts (6), posing an economic burden through prolonged hospitalization, reduced quality of life, and increased healthcare costs (6). The rising incidence of CDI highlights the urgent need for effective preventive strategies to reduce infection rates and improve patient outcomes.

Probiotics have gained attention for their role in regulating gut microbiota (7, 8). The World Health Organization (WHO) defines probiotics as live microorganisms that confer health benefits when administered in adequate amounts (9, 10). Probiotics are thought to compete with pathogens, helping to maintain gut microbiota balance, promote gut barrier function, preserve intestinal integrity, and modulate immune responses (11, 12). Consequently, several meta-analyses have investigated the effectiveness of probiotics in preventing CDI.

Despite promising findings, some studies have reported no significant effect of probiotics on CDI prevention. These inconsistencies may stem from variations in probiotic strain, dosage, and patient population, which can influence the risk of diarrhea and CDI. Therefore, evaluating the effect of probiotics on CDI prevention using both risk ratios (RR) and odds ratios (OR) in a strain-specific manner is crucial. This underscores the importance of synthesizing the available evidence to clarify the efficacy of probiotic supplementation in preventing CDI.

## 2 Methods

This umbrella review of systematic reviews and meta-analyses was conducted in accordance with the PRISMA 2020 guidelines (13). The study protocol was registered in PROSPERO (ID CRD420251062730).

### 2.1 Search strategy

A comprehensive systematic search was conducted in PubMed, Scopus, Web of Science, Embase, and the Cochrane Library from database inception through December 2025. Reference lists of relevant studies were also screened to identify additional articles. The search strategy was developed using a combination of MeSH terms and keywords (Supplementary Table 1). Moreover, the search was restricted to articles published in the English language.

### 2.2 Inclusion and exclusion criteria

This study integrated the systematics reviews and meta-analyses investigating the impact of probiotics in prevention of CDI, specifically those that provided effect sizes (ESs) along with their respective confidence intervals (CIs). The PICO criteria for this umbrella meta-analysis were defined as follows: population: individuals of all ages, both below and above 18 years receiving probiotic treatment; intervention: administration of probiotics; comparison: a control or placebo group; and outcome: prevention of CDI. The CDI outcome definition and diagnostic timeframe (e.g., during antibiotic therapy or within 4–8 weeks’ post-therapy) were accepted as defined by each included systematic review. *In vitro*, *in vivo*, *ex vivo* studies, case reports, observational studies, and quasi-experimental studies were excluded.

### 2.3 Methodological quality assessment, overlap, and grading of the evidence

The Measurement Tool to Assess Systematic Reviews (AMSTAR)2 questionnaire was used to qualify the included studies by two independent researchers (14), which consists of 16 items rated as “yes,” “no,” or “not applicable.” Any disagreement was resolved via discussing with third investigator. To address overlap of primary studies across included systematic reviews, the Corrected Covered Area (CCA) was calculated. For outcomes reported by multiple reviews, a single primary systematic review was selected for synthesis based on pre-specified criteria: 1) highest AMSTAR-2 confidence rating, 2) most recent publication date, and 3) largest number of included primary trials. Sensitivity analyses were conducted by sequentially excluding non-primary reviews to assess the robustness of the umbrella review estimates. The overall certainty of evidence for the main outcome (CDI prevention) was evaluated using the GRADE approach, considering the quality of the included reviews and the underlying primary studies. The quality of evidence was categorized into four categories based on evaluation criteria, i.e., high, moderate, low, and very low (15).

### 2.4 Study selection and data extraction

The screening process was completed by two independent reviewers in accordance with the predefined eligibility criteria. Initially, the titles and abstracts of the articles were evaluated. Subsequently, the full-texts of the eligible articles were evaluated to determine their eligibility for inclusion in the current umbrella meta-analysis. The extracted data included the name of the first author, year of publication, the geographical location, number of included studies in each meta-analysis, total sample sizes and ESs and CIs of outcomes.

### 2.5 Data synthesis and statistical analysis

The pooled ES and its associated 95% CI were estimated using random-effects models performed using restricted maximum likelihood (REML) model (16). ESs are reported as RR or OR with 95% CI. The *I*^2^ index was used to evaluate the heterogeneity within the meta-analysis and a significant level of heterogeneity in the data was established when *I*^2^ exceeded 50% (16). Pre-planned subgroup analyses were conducted for clinically relevant strata: probiotic type (e.g., *Saccharomyces boulardii*, *Lactobacillus-based*, multi-strain), sample size, and age group (adult, pediatric). Where sufficient data were available, pooled estimates were calculated for each subgroup using a random-effects model, and tests for subgroup differences were performed. A sensitivity analysis was performed to evaluate the impact of excluding a specific study on the overall ES. All statistical analyses were conducted using STATA version 16.0 (Stata Corporation, College Station, TX, US). A *p*-value of less than 0.05 was considered significant.

## 3 Results

### 3.1 Study selection and study characteristics

**Figure 1:**
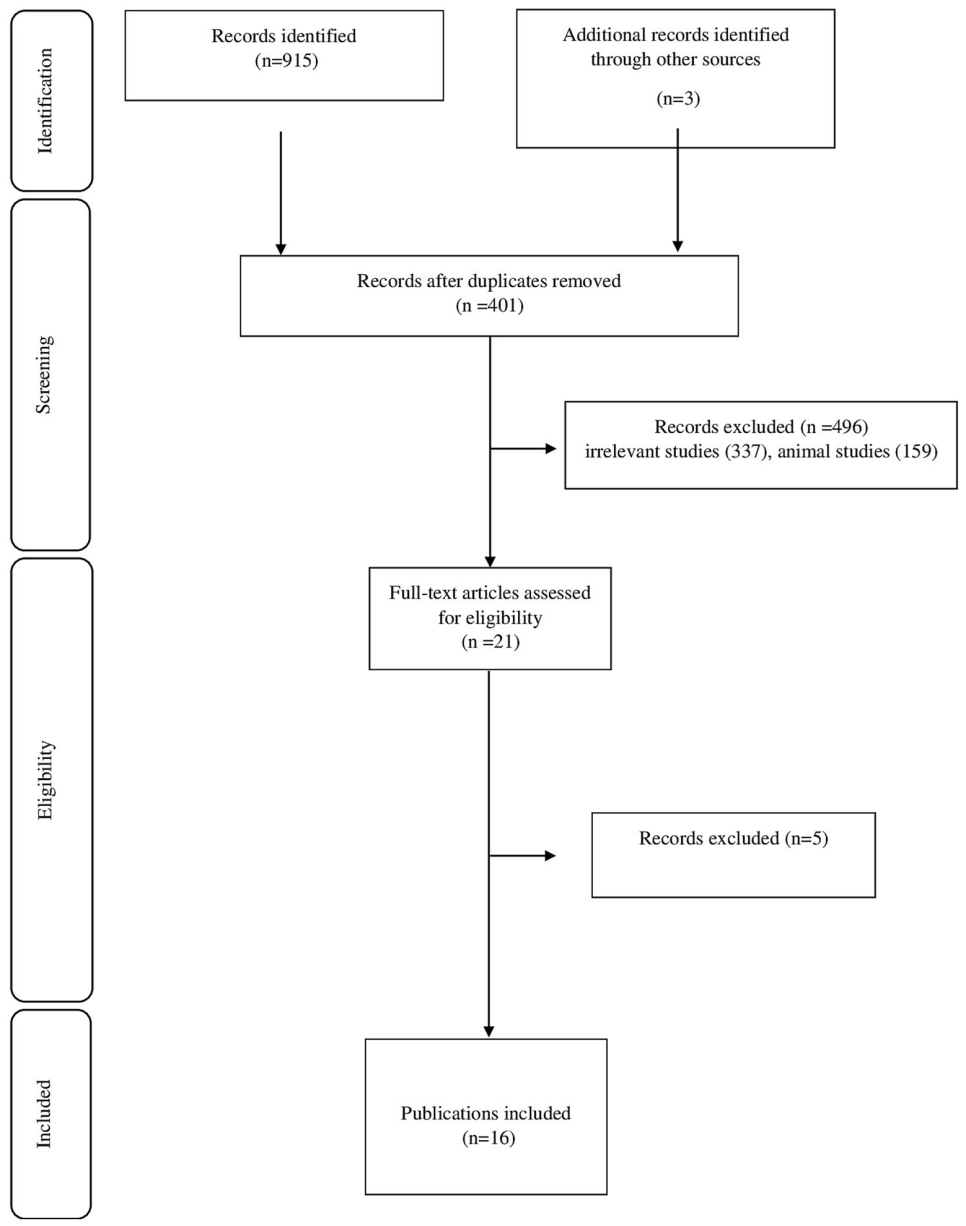
PRISMA flow diagram.

There were 918 records found in the database search. 401 duplicate studies were eliminated, leaving 517 distinct studies for screening. After 496 of these were disqualified on the basis of abstract and title review, 21 articles remained for full-text assessment. A thorough evaluation led to the exclusion of five studies. 16 studies ultimately met the predetermined inclusion criteria. Figure 1 shows the selection procedure for the study. In the present review, a total of 16 systematic reviews were included (Table 1) (17–32). The CCA score was 15%, indicating “slight” overlap. For the primary outcome of CDI prevention based on RR, the review by Johnston et al. (2012) was selected as the primary synthesis due to its comprehensive scope and methodological rigor. All these studies were published between 2005 up to 2025. The most used probiotics were *Saccharomyces, Lactobacillus, Bifid bacterium, Streptococcus Clostridium* and mix of probiotics.

**Table 1:** Study characteristics of included studies.

Citation (First author et al., year)	No. of studies in meta-analysis	Location	No. of participants in meta-analysis	Intervention	Quality assessment scale and outcome
Avadhani A et al. 2010	4	Australia	471	*Saccharomyces*. *Lactobacillus. Bifid bacterium*	Yes (JBI’s assessment)
Johnston BC et al. 2012	20	Canada	3,818	*Saccharomyces. Lactobacillus. Bifid bacterium. Streptococcus*	Yes (Cochrane) 5/20 low quality
Salari P et al. 2012	19	Iran	3,867	*Saccharomyces. Lactobacillus. Bifid bacterium. Streptococcus*	Yes (Jadad score) 3/19 low quality
Pattani R et al. 2013	9	Canada	1,107	*Lactobacillus. Saccharomyces. Streptococcus*	Yes (Cochrane) 4/9 low quality
McFarland VL et al. 2013	5	US	1,211	*Saccharomyces. Lactobacillus. Bifid bacterium*	Yes (Cochrane) 4/5 low quality
McFarland VL et al. 2015	23	US	4,476	*Saccharomyces. Lactobacillus. Bifid bacterium*	Yes (Cochrane) 3/24 low quality
Lau CSM et al. 2016	26	US	7,957	*Saccharomyces. Lactobacillus. Bifid bacterium. Streptococcus*	Yes (Cochrane) 2/26 low quality
Shen NT et al. 2017	18	US	6,139	*Saccharomyces. Lactobacillus. Bifid bacterium. Streptococcus*	Yes (Cochrane) 10/18 low quality
Vernaya M et al. 2017	5	US	3,461	*Bifidobacteriaum. Lactobacillus. Saccharomyces*	Yes (Cochrane) 0/5 low quality
Johnston BC et al. 2018	13	Canada	5,074	Saccharomyces. Lactobacillus. Bifid bacterium. Streptococcus	Yes (Cochrane) 7/13 low quality
Ma Y et al. 2019	11	China	4,523	Saccharomyces. Lactobacillus. Bifid bacterium. Streptococcus	Yes (Cochrane) 2/11 low quality
Goodman C et al. 2021	42	Australia	11,305	Saccharomyces. Lactobacillus. Bifid bacterium. Streptococcus	Yes (Cochrane) 5/42 high quality
Raseen Tariq et al. 2023	6	US	1,049	*Saccharomyces boulardi*	Yes (Cochrane) 2/6 high
Dabrowski V et al. 2023	10	Ireland	5,146	*Lactobacillus. Bifid bacterium. Streptococcus. Clostridium*	Not included in meta-analysis
Almutawif YA et al. 2025	4	Saudi Arabia	9,226	*Probiotic yogurt drink, Danactive. S. boulardii. L. casei. Lyophilized microbial cells*	Yes (Cochrane) 1/4 low quality
Wanyama H et al. 2025	15	United Kingdom	7,427	*Lactobacillus. Bifid bacterium. Streptococcus. Clostridium*	Yes (Cochrane) 3/15 low quality

NR, Not reported.

### 3.2 Quality assessment and certainty of evidence

The methodological quality of the included reviews, assessed using AMSTAR-2, is detailed in Table 2. Eleven of the 16 reviews were rated as having “critically low” overall confidence, primarily due to shortcomings in protocol registration, risk of bias assessment, and reporting of excluded studies (17–19, 21–23, 25–28, 31). Using the GRADE framework, the overall certainty of evidence for the efficacy of probiotics in preventing CDI was assessed as low, downgraded due to the methodological limitations of the included reviews and the indirectness introduced by pooling diverse probiotic strains and populations.

**Table 2:** Assessment of the methodological quality of meta-analysis.

Study (Author, Year)	Q1	Q2	Q3	Q4	Q5	Q6	Q7	Q8	Q9	Q10	Q11	Q12	Q13	Q14	Q15	Q16	Overall confidence
Almutawif YA et al. 0.2025	Yes	No	Partial Yes	Partial Yes	Partial Yes	Partial Yes	No	Yes	Yes	No	Partial Yes	Partial Yes	Partial Yes	Partial Yes	Partial Yes	Yes	Critically low
Avadhani A et al. 2010	Yes	No	No	Partial Yes	No	No	No	Partial Yes	Partial Yes	No	Partial Yes	No	No	No	Partial Yes	No	Critically low
Dabrowski V et al. 2023	Yes	No	Partial Yes	Partial Yes	Partial Yes	Partial Yes	No	Yes	Yes	No	Yes	Partial Yes	Partial Yes	Partial Yes	Partial Yes	Yes	Critically low
Goodman C et al. 2021	Yes	Partial Yes	Yes	Yes	Yes	Yes	Partial Yes	Yes	Yes	Yes	Yes	Yes	Yes	Yes	Partial Yes	Yes	Moderate
Johnston BC et al. 2018	Yes	No	Yes	Yes	Yes	Yes	No	Yes	Yes	Yes	Yes	Yes	Yes	Yes	Yes	Yes	Critically low
Johnston BC et al. 2012	Yes	No	Yes	Yes	Partial Yes	Partial Yes	No	Yes	Yes	No	Yes	Partial Yes	Partial Yes	Yes	Partial Yes	Yes	Critically low
Lau CSM et al. 2016	Yes	No	Partial Yes	Partial Yes	No	No	No	Partial Yes	Partial Yes	No	Partial Yes	No	No	Partial Yes	Partial Yes	No	Critically low
Ma Y et al. 2019	Yes	Yes	Yes	Yes	Yes	Yes	Partial Yes	Yes	Yes	Yes	Yes	Yes	Yes	Yes	Yes	Yes	High
McFarland VL et al. 2015	Yes	No	Yes	Partial Yes	Partial Yes	Partial Yes	No	Yes	Partial Yes	No	Partial Yes	Partial Yes	Partial Yes	Partial Yes	Partial Yes	Yes	Critically low
McFarland VL et al. 2013	Yes	No	No	Partial Yes	No	No	No	Partial Yes	No	No	No	No	No	No	No	No	Critically low
Pattani R et al. 2013	Yes	No	Partial Yes	Partial Yes	No	No	No	Partial Yes	Partial Yes	No	Partial Yes	Partial Yes	Partial Yes	Partial Yes	Partial Yes	No	Critically low
Salari P et al. 2012	Yes	No	No	Partial Yes	No	No	No	Partial Yes	No	No	No	No	No	No	No	No	Critically low
Shen NT et al. 2017	Yes	No	Yes	Yes	Yes	Yes	Partial Yes	Yes	Yes	Yes	Yes	Yes	Yes	Yes	Yes	Yes	Low
Tariq R et al. 2023	Yes	Yes	Yes	Yes	Yes	Yes	Partial Yes	Yes	Yes	Yes	Yes	Yes	Yes	Yes	Yes	Yes	High
Vernaya M et al. 2017	Yes	No	Partial Yes	Partial Yes	No	No	No	Partial Yes	Partial Yes	No	Partial Yes	Partial Yes	Partial Yes	Partial Yes	Partial Yes	No	Critically low
Wanyama H et al. 2025	Yes	Yes	Yes	Yes	Yes	Yes	Partial Yes	Yes	Yes	Yes	Yes	Yes	Yes	Yes	Yes	Yes	High

### 3.3 Probiotics supplementation in prevention of CDI based on relative risk analysis

**Figure 2:**
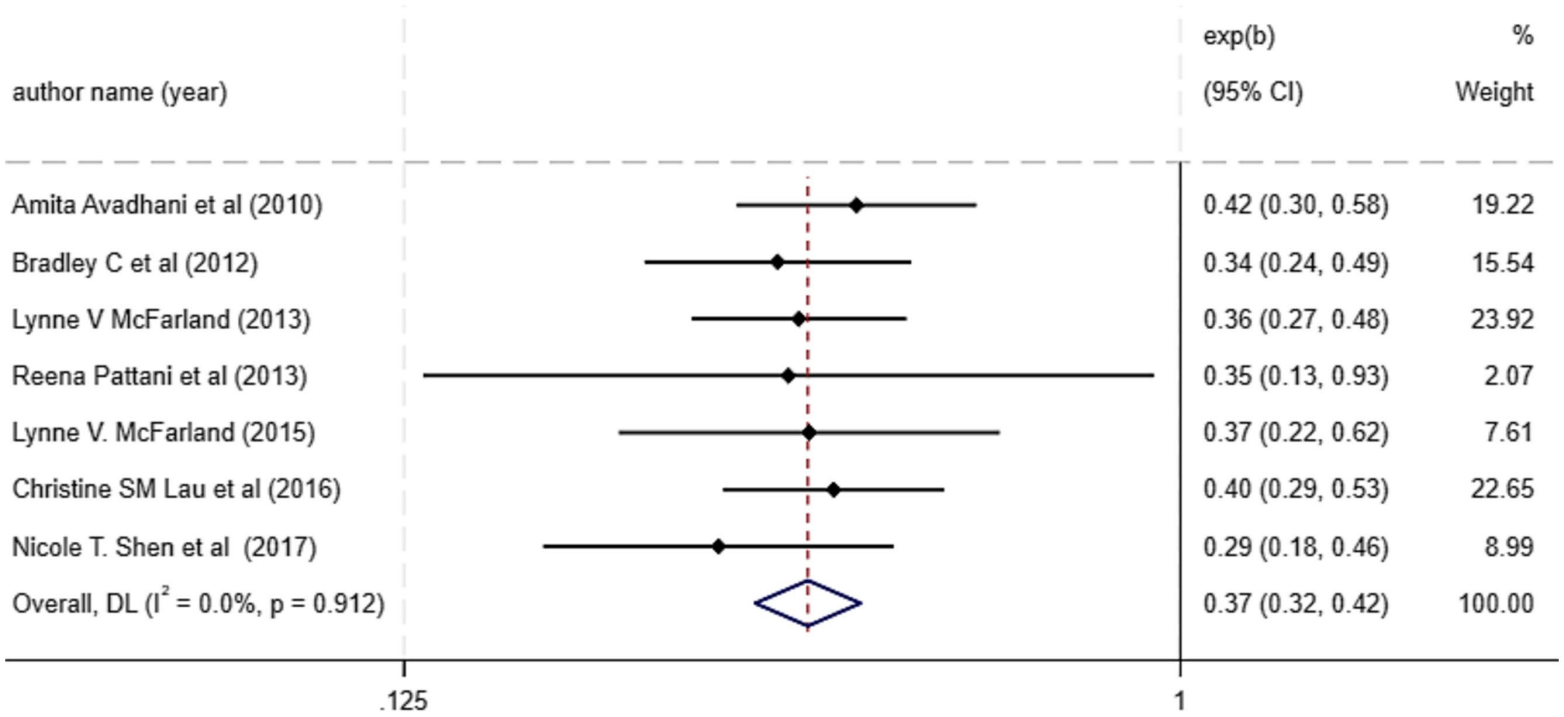
Effect size and 95% CIs presented in forest plot of the studies on the probiotics supplementation in CDI prevention based on RR analysis. Weights are from random-effects model.

The primary pooled estimate, derived from seven meta-analyses reporting RR, indicated that probiotic supplementation significantly reduced the risk of CDI by 63% (RR = 0.37; 95% CI: 0.32 to 0.42; *p* < 0.001) with no statistical heterogeneity (I^2^ = 0%) (Figure 2). Our subgroup analysis showed that, probiotics are effective in preventing CDI in studies less than 5,000 participants. Significant reductions in CDI risk were observed for Saccharomyces boulardii (RR = 0.38; 95% CI: 0.27 to 0.53; 3 reviews) and multi-strain formulations (RR = 0.34; 95% CI: 0.24 to 0.49; 4 reviews). The test for subgroup differences between single-strain and multi-strain probiotics was not significant (Table 3). Moreover, sensitivity analysis indicated that exclusion of any single study did not affect the overall pooled effect size (Supplementary Figure 1). Furthermore, Bag’s test revealed no evidence of publication bias (*p* = 0.999).

**Table 3:** Overall and subgroup analyses.

Outcomes	Subgroups	Studies	Test of effect	Test of heterogeneity	
			ES (95%CI)	I^2^ (%)	*P*
CDI prevention	Overall	7	0.37 (0.32, 0.42)	0.0	0.912
Type of strains	S, L, B, St	3	0.35 (0.29, 0.44)	0.0	0.679
	S, L, B	3	0.38 (0.31, 0.47)	0.0	0.774
	S, L, St	1	0.35 (0.13, 0.93)	-	-
Sample size	≥ 5,000 participants	2	0.36 (0.27, 0.47)	16.2	0.275
	<5,000 participants	5	0.37 (0.31, 0.44)	0.0	0.930

L; Lactobacillus, B; Bifid bacterium, St; Streptococcus, S; Saccharomyces.

### 3.4 Probiotics supplementation in prevention of CDI based on odds ratio analysis

**Figure 3:**
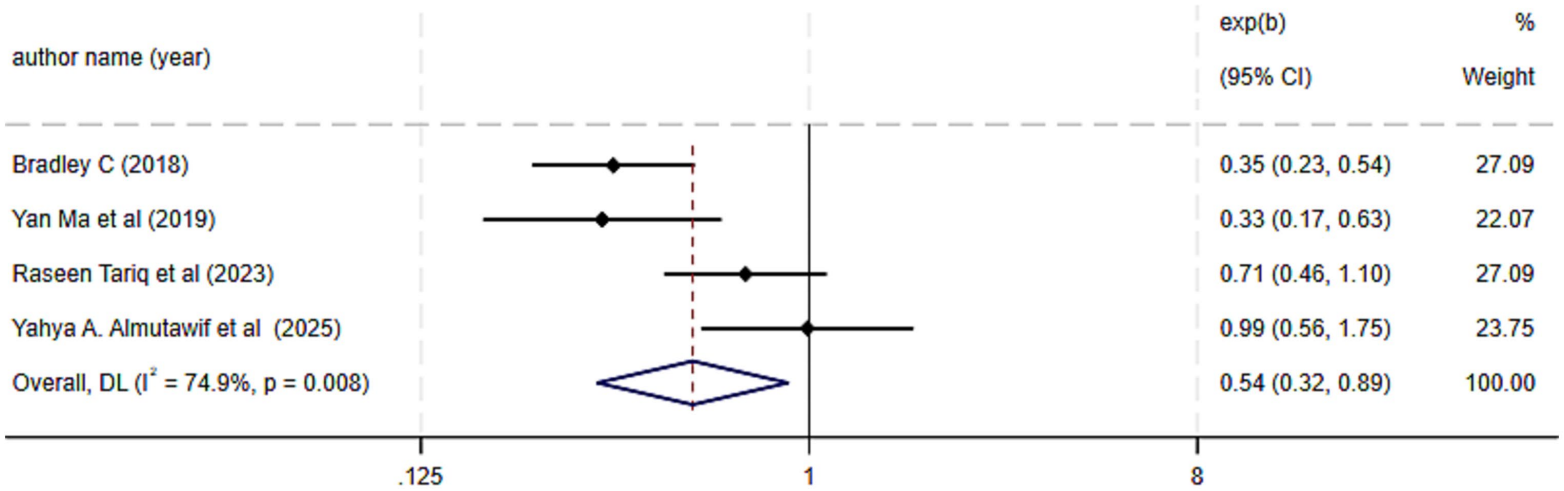
Effect size and 95% CIs presented in forest plot of the studies on the probiotics supplementation in CDI prevention based on OR analysis. Weights are from random-effects model.

The pooled effect size of four meta-analyses, revealed that probiotic intervention significantly reduced the odds of CDI prevention by 46% compared to the control group (OR = 0.54; 95% CI: 0.32 to 0.89, *p* = 0.001), with substantial heterogeneity (I^2^ = 74.9%, P-heterogeneity = 0.008) (Figure 3). This finding complements the RR analysis but must be interpreted within the context of this heterogeneity. Sensitivity analysis indicated that the overall effect size was robust, with no single study exerting influence (Supplementary Figure 2). Furthermore, no evidence for publication bias was seen based on Bag’s tests (*p* = 0.343).

### 3.5 Probiotic supplementation on CDI-associated diarrhea

The evidence for CDI-associated diarrhea was mixed. One meta-analysis found no significant effect of *Bifid bacterium*, *Lactobacillus*, and *Saccharomyces* supplementation in elderly patients (OR = 0.66; 95% CI: 0.26 to 1.66) (31). However, another meta-analysis reported that mixed probiotic formulations reduced the incidence of CDI-associated diarrhea by approximately 40% in adult patients (RR = 0.60; 95% CI: 0.43 to 0.83) (32). A meta-analysis similarly demonstrated a significant reduction in acute diarrhea following probiotic supplementation (WMD = −0.65; 95% CI: −0.94 to −0.38) (28). The probiotic *Lactobacillus* was associated with a 75% reduction in the risk of CDI-associated diarrhea compared with placebo (RR = 0.25; 95% CI: 0.08 to 0.47) (33).

### 3.6 Safety and adverse events

Twelve of the sixteen included reviews reported on safety. The most commonly reported adverse events were mild gastrointestinal symptoms (e.g., bloating, flatulence). None of the reviews found a statistically significant increase in serious adverse events attributable to probiotic supplementation compared to placebo or control.

## 4 Discussion

This umbrella review synthesizes evidence from systematic reviews and meta-analyses on the efficacy of probiotics in preventing CDI. Our primary analysis demonstrates a significant reduction in CDI risk following probiotic supplementation (RR = 0.37). The absence of statistical heterogeneity (I^2^ = 0%) suggests a consistent effect across the included reviews and diverse populations. These findings suggest that probiotics may be considered as effective strategy to prevent the risk of CDI specifically in high-risk population such as hospitalized patients. Probiotics are more potent to restore the gut microbiota balance which may contribute to the CDI (34, 35). Also, they are effective in strengthening intestinal barrier integrity (12), and modulating immune responses (36–38). While most previous meta-analyses align with our pooled result, one review by Dabrowski et al. reported no significant benefit for patients exposed to antibiotics (19, 39, 40).

Subgroup analyses provided further insight. Strain-specific analyses indicated that multi-strain probiotics and those containing *Saccharomyces boulardii* had pronounced effects. This highlights the importance of probiotic composition. Synergistic effects may arise from the combined actions of different strains: *Saccharomyces* is noted for immunomodulation, *Lactobacillus* for restoring microbial balance (41, 42), and *Bifid bacterium* for supporting barrier function (43). Furthermore, the beneficial effect was consistent across studies of varying sample sizes, supporting the generalizability of the findings. Similarly, our meta-review demonstrated probiotics reduce the odds of CDI as evidenced by OR. This finding complements the abovementioned RR results and can be recommended in clinical setting for CDI prevention reliably.

This umbrella review has several strengths, including a comprehensive search, formal assessment of overlap, and dual appraisal of methodological quality (AMSTAR-2) and evidence certainty (GRADE). The consistent effect across metrics (RR and OR) and low heterogeneity for the primary RR analysis suggest a robust signal. Furthermore, the beneficial effects of probiotics were observed across studies of varying sample sizes, which may support the generalizability of the findings. However, significant limitations must be acknowledged. The majority of included systematic reviews were of critically low methodological quality, which indirectly lowers confidence in our umbrella review estimates. The low GRADE certainty rating further tempers the strength of any conclusions. We were unable to perform robust quantitative analyses for all pre-specified clinical strata (e.g., specific dosage, patient sub-populations) due to inconsistent reporting in the source reviews. Therefore, the findings should be interpreted with caution.

## 5 Conclusion

This umbrella review of systematic reviews and meta-analyses suggests that probiotic supplementation may be associated with a reduced risk of CDI, as indicated by both RR and OR estimates. The most consistent evidence supports the use of *Saccharomyces boulardii* and multi-strain formulations. However, due to the predominantly low methodological quality of the underlying systematic reviews, these conclusions should be interpreted with caution. High-quality, sufficiently powered primary trials and subsequent rigorous systematic reviews are needed to establish reliable, strain-specific, and dose-specific recommendations for clinical practice.

## Data Availability

The datasets presented in this study can be found in online repositories. The names of the repository/repositories and accession number(s) can be found in the article/Supplementary material.
