# Supplementary material for: Efficacy of probiotic supplementation in preventing Clostridioides difficile infection: an umbrella review of systematic reviews and meta-analysis
Source: Front Nutr. 2026 Mar 9;13:1699223. doi: 10.3389/fnut.2026.1699223 (PMC13006325; doi:10.3389/fnut.2026.1699223)

**Supplementary Table 1.** Search strategy

("Clostridioides difficile"[Mesh] OR "Clostridium difficile"[tiab] OR "C. difficile"[tiab] OR "CDI"[tiab] OR "Clostridioides difficile infection"[tiab]) AND ("Probiotics"[Mesh] OR probiotic[tiab] OR probiotics[tiab] OR "Lactobacillus"[tiab] OR "Bifidobacterium"[tiab] OR "Saccharomyces"[tiab] OR "synbiotics"[tiab]) AND ("prevention"[tiab] OR "preventive"[tiab] OR "prophylaxis"[tiab] OR "risk reduction"[tiab]) AND ("Systematic Reviews "[tiab] OR " Meta-Analyses "[tiab]).

**Supplementary Figure 1**. Sensitivity analysis for probiotics supplementation in CDI prevention based on RR analysis


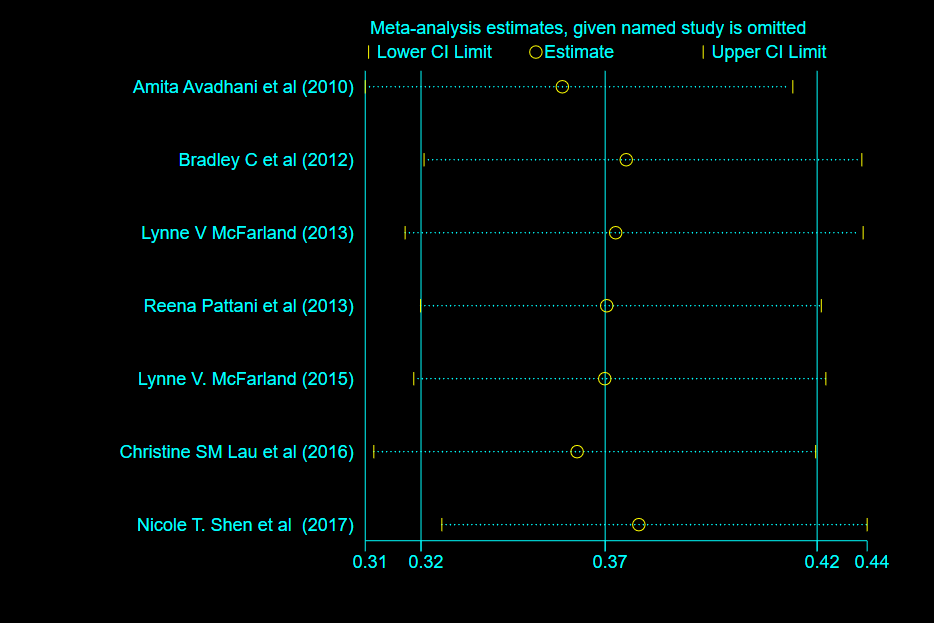


**Supplementary Figure 2.** Sensitivity analysis for probiotics supplementation in CDI prevention based on OR analysis


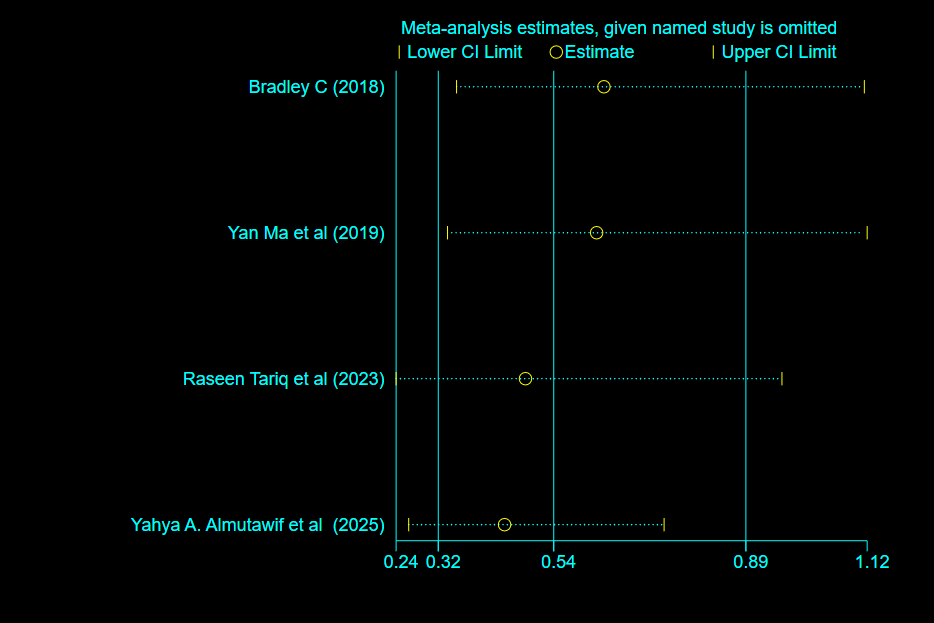

Supplement: Supplementary file 1 [file Table_1.docx]
